# Effect of Silica Fume and Fly Ash Admixtures on the Corrosion Behavior of AISI 304 Embedded in Concrete Exposed in 3.5% NaCl Solution

**DOI:** 10.3390/ma12234007

**Published:** 2019-12-03

**Authors:** Miguel Angel Baltazar-Zamora, David M. Bastidas, Griselda Santiago-Hurtado, José Manuel Mendoza-Rangel, Citlalli Gaona-Tiburcio, José M. Bastidas, Facundo Almeraya-Calderón

**Affiliations:** 1 Facultad de Ingeniería Civil-Xalapa, Universidad Veracruzana, Lomas del Estadio S/N, Zona Universitaria, C.P. 91000 Xalapa, Veracruz, Mexico; 2 National Center for Education and Research on Corrosion and Materials Performance, NCERCAMP-UA, Dept. Chemical, Biomolecular, and Corrosion Engineering, The University of Akron, 302 E Buchtel Ave, Akron, OH 44325-3906, USA; dbastidas@uakron.edu; 3 Facultad de Ingeniería Civil—Unidad Torreón, UADEC, C.P. 27276 Torreón, Mexico; grey.shg@gmail.com; 4 FIC., Universidad Autónoma de Nuevo León, Ave. Pedro de Alba S/N, Ciudad Universitaria, C.P. 66455 San Nicolás de los Garza, Mexico; jmmr.rangel@gmail.com; 5 FIME—CIIIA, Universidad Autónoma de Nuevo León, Av. Universidad S/N, Ciudad Universitaria, C.P. 66455 San Nicolás de los Garza, Mexico; citlalli.gaona@gmail.com (C.G.-T.); falmeraya.uanl.ciiia@gmail.com (F.A.-C.); 6 National Centre for Metallurgical Research (CENIM), CSIC, Ave. Gregorio del Amo 8, 28040 Madrid, Spain; bastidas@cenim.csic.es

**Keywords:** concrete, concrete admixtures, corrosion, marine environment, silica fume, fly ash

## Abstract

The use of supplementary cementitious materials such as fly ash, slag, and silica fume improve reinforced concrete corrosion performance, while decreasing cost and reducing environmental impact compared to ordinary Portland cement. In this study, the corrosion behavior of AISI 1018 carbon steel (CS) and AISI 304 stainless steel (SS) reinforcements was studied for 365 days. Three different concrete mixtures were tested: 100% CPC (composite Portland cement), 80% CPC and 20% silica fume (SF), and 80% CPC and 20% fly ash (FA). The concrete mixtures were designed according to the ACI 211.1 standard. The reinforced concrete specimens were immersed in a 3.5 wt.% NaCl test solution to simulate a marine environment. Corrosion monitoring was evaluated using the corrosion potential (*E*_corr_) according to ASTM C876 and the linear polarization resistance (LPR) according to ASTM G59. The results show that AISI 304 SS reinforcements yielded the best corrosion behavior, with *E*_corr_ values mainly pertaining to the region of 10% probability of corrosion, and corrosion current density (*i*_corr_) values indicating passivity after 105 days of experimentation and low probability of corrosion for the remainder of the test period.

## 1. Introduction

Due to its relatively low cost and high compressive resistance capability, reinforced concrete is frequently used as a construction material worldwide. However, several aggressive agents found in the environment degrade steel reinforcements, cement, and concrete [1]. Corrosion of steel embedded in concrete was extensively studied since the 1950s, and, in the last 30 years, research concentrated on minimizing the steel corrosion rate. Researchers studying this phenomenon reported a great number of different approaches. Current trends focus on innovation in concrete technology, finding alternative materials to composite Portland cement (CPC), the use of different reinforcing steels, the use of corrosion inhibitors, and the impact of the exposure environment, such as marine or urban, both natural and simulated [2,3,4,5,6].

According to the literature, the external, non-structural causes that usually affect the durability of concrete structures are mainly a result of their exposure and service conditions. The service lifetime of a reinforced concrete structure can be reduced by corrosion of the embedded reinforcing steel due to aggressive agents from the environment [7]; one of the main causes is chloride ions. Steel rebars inside reinforced concrete structures (RCS) are susceptible to corrosion when the permeation of chloride from de-icing salts, marine aerosols, or sea water, if they are fully or partially submerged, exceeds a chloride threshold level (CTL) at the steel surface [8,9]. In the case of Mexico, data that can give an idea of losses due to corrosion are unfortunately not available, yet this country has more than 10,000 km of coastline where there are many reinforced concrete structures susceptible to corrosion damage. However, the total direct cost of corrosion in the United States (US) was determined to be $276 billion per year, which is 3.1% of the US gross domestic product (GDP) [10].

Supplementary cementitious materials (SCMs) such as silica fume (SF), fly ash (FA), and rice husk ash (RHA) are commonly used in concrete formulations around the world [11,12] for different economic and environmental reasons. Moreover, FA particles react with calcium hydroxide to produce hydration products that strongly decrease the concrete porosity [13]. Another type of waste by-product additive exhibiting good results is blended, lime-stabilized drilling mud and cement [14,15,16]. The recycling of these products in concrete production has positive environmental effects, minimizing problems associated with their disposal [17]. In the last 20–30 years, sugar-cane bagasse ash (SCBA), an agro-industrial waste by-product, was used with great success as a partial substitute of **CPC in concrete** and showed benefits against corrosion [18,19].

Silica fume was in use in the concrete industry for over 20 years. The silica fume reacts with calcium hydroxide in the presence of water to form cementitious compounds consisting of calcium silicate hydrate. The incorporation of silica fume in concrete improves its strength and durability characteristics. It is also reported that silica fume was successfully used to produce chemically resistant concrete with very high strength and low permeability. Several researchers showed that the addition of silica fume significantly reduces permeability [20]; however, an investigation reported that the use of 10% silica fume as cement replacement material in producing 70-MPa concrete can have a beneficial effect in terms of reduced corrosion rate. Adverse effects may result from using silica fume at a higher replacement level of 20% [21].

The use of an industrial waste by-product as a pozzolanic additive in concrete structures, mainly the use of fly ash (FA), was studied extensively around the world. FA is a by-product of the combustion of coal in thermal power plants and is gathered by electrostatic precipitators from the combustion gases before they are discharged into the atmosphere. Only flying particles produced from the burning of the coal are attracted to the precipitators. Most of the FA is formed by silica- and alumina-rich particles with only a small amount of unburnt coal particles. As a result, FA reacts effectively with the concrete portlandite to form more dense and resistant cementitious products, improving in this manner the long-term mechanical and durability properties of concrete [22].

Results on FA research showed that its presence in concrete improves the workability of mortars and concretes in their fresh state. In their hardened state, the presence of FA also improves structural properties such as compressive strength; however, this improvement occurs at a later stage than in the mortars and concretes without FA [23].

The aim of this work was to study the corrosion behavior of reinforced concrete made with partial replacement of CPC, using 20% SF or 20% FA and the remaining 80% CPC. A reinforced concrete made with 100% CPC was used as a control sample. Two types of steel reinforcements were studied, conventional AISI 1018 carbon steel (CS) and austenitic AISI 304 stainless steel (SS). The reinforced concrete specimens were immersed in a 3.5% NaCl solution for up to 365 days. 

## 2. Materials and Methods

Three different concrete mixtures manufactured using type 30R CPC according to the NMX C 414 standard [24] and partial replacement of CPC by SF or FA industrial waste by-products were studied. The first concrete mixture was made with 100% CPC, the second contained 80% CPC and 20% SF, and the third contained 80% CPC and 20% FA. The concrete mixtures were designed according to the ACI 211.1 standard [25] to obtain a compressive strength of F’c = 35.7 MPa. To determine the compressive strength of the three concrete mixtures evaluated in the present investigation, cylindrical specimens of 15 cm in diameter and 30 cm in height were used, and the test was performed according to the NMX-C-083-ONNCCE-2014 standard [26]. The tests to determine the physical characteristics of the aggregates used to manufacture the concrete mixtures were performed according to the following ASTM standards: ASTM C-127-15 (Standard Test Method for Relative Density (Specific Gravity) and Absorption of Coarse Aggregate) [27], ASTM C-128-15 (Standard Test Method for Relative Density (Specific Gravity) and Absorption of Fine Aggregate) [28], ASTM C29/C29M–07 (Standard Test Method for Bulk Density (Unit Weight) and Voids in Aggregate) [29], and ASTM C33/C33M–16e1 (Standard Specification for Concrete Aggregates) to determine the fineness modulus and maximum aggregate size [30]. All of the results of the aforementioned tests are required to meet the ACI 211.1 standard.

Table 1 details the physical characteristics of the aggregates, and Table 2 shows the amounts of cement, water, and aggregates used in each of the three different concrete mixtures, obtained according to the ACI standard.

### 2.1. Characterization of Fresh and Hardened Concrete

Physical and mechanical characterization of the fresh and hardened concrete mixtures was performed according to the ASTM C 1064 standard [31], and the NMX C 156 [32], NMX C 162 [33], and NMX C 083 [26] standards. Table 3 shows the results obtained for the three studied mixtures.

### 2.2. Concrete Specimens

Prismatic concrete specimens with dimensions of 15.0 × 12.0 × 7.0 cm were manufactured with two rebars embedded in the concrete. Table 4 shows the chemical composition of the AISI 1018 CS and AISI 304 SS, both of which were 15 cm in length and 0.95 mm in diameter. Each as-received rebar was partly coated with an anticorrosive paint in order to leave a length of 5 cm exposed to the concrete environment. The concrete was made with a water-to-cement ratio of 0.50. The first two specimens were made with 100% CPC 30R (denoted 4AN (control specimen) and 4AI); the second two specimens were made replacing 20% by weight of CPC 30R with SF (denoted 4BN and 4BI); the third two specimens were made replacing 20% by weight of CPC 30R with FA (denoted 4CN and 4CI). The specimens were cured according to the NMX-C-159 standard [34] by immersion in water for 27 days. After the curing period, the six reinforced concrete specimens were immersed in a 3.5% by weight NaCl solution for 365 days, simulating a marine environment, and they were then subjected to electrochemical tests.

As indicated above, the nomenclature used to perform the analysis of the results of *E_corr_* and *i_corr_* was made up of three characters; the first two characters indicate the type of studied concrete mix, and the third character refers to the reinforcing steel embedded in the concrete mix. The nomenclature used in the present study is as follows:

- 4A indicates the concrete mix with 100% CPC;
- 4B indicates the concrete mix with 80% CPC + 20% SF;
- 4C indicates the concrete mix with 80% CPC + 20% FA;
- N indicates rebars of AISI 1018 carbon steel;
- I indicates rebars of AISI 304 stainless steel.

### 2.3. Electrochemical Techniques

**Figure 1:**
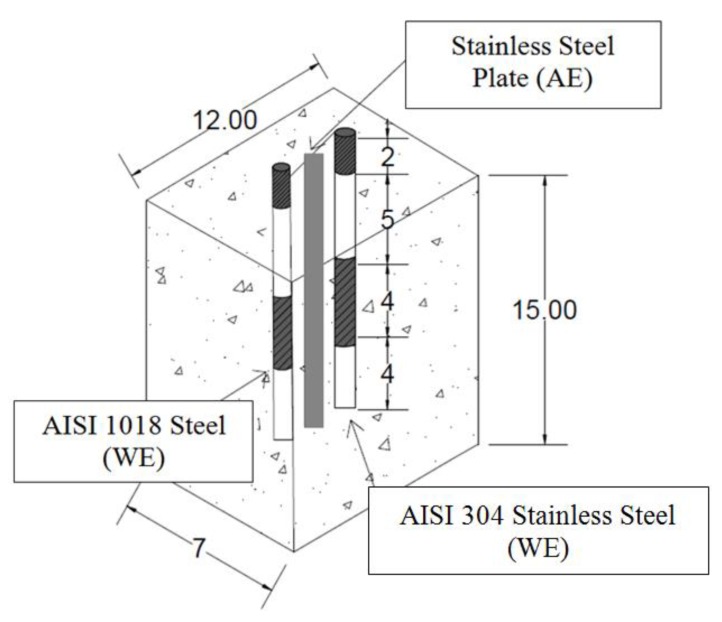
Illustration of the tested specimens: three-electrode corrosion cell and experimental arrangement.

A conventional three-electrode cell configuration was used for electrochemical studies. The AISI 1018 CS and AISI 304 SS were used as the working electrode. A standard copper/copper sulfate (Cu/CuSO_4_, CSE) and AISI 316 SS plate were used as the reference and counter/auxiliary electrodes (CE or AE), respectively, see Figure 1. Electrochemical measurements were carried out using a Gill-AC potentiostat/galvanostat/ZRA (ACM Instruments, Cark In Cartmel, UK). The linear polarization resistance (LPR) measurements were recorded using a potential sweep rate of 10 mV/min at a potential scan range between −20 and +20 mV, according to the ASTM G59-97 standard [35]. The half-cell corrosion potential (*E*_corr_) was recorded according to the ASTM C876-15 standard [36]. Corrosion rate (CR) was calculated from the corrosion current density (*i*_corr_) using the LPR results [37]. 

Corrosion experiments were performed by immersion in a 3.5 wt.% NaCl solution at 25 °C.

Corrosion monitoring (*E*_corr_ and *i*_corr_) was conducted weekly for the reinforced concrete specimens immersed in the 3.5 wt.% NaCl solution at room temperature, and the measurements were performed in triplicate. The results were analyzed using Version 4 Analysis specialized software (ACM Instruments, Cark In Cartmel, United Kingdom).

As previously indicated by the authors [38,39,40], the *i*_corr_ and the CR were estimated from the LPR method using the Stern and Geary equation (Equation (1)).

(1) $$i_{corr}=\frac{B}{R_{p}},$$

where B is a constant, equal to 26 mV for active rebar corrosion [41]. 

To assess the degree of corrosion of the reinforced concrete specimens, the *E*_corr_ parameter was used in accordance with the ASTM C 876-15 standard [36], which establishes the criteria that relate the *E*_corr_ with the probability of corrosion for the CPC/AISI 1018 CS system, as shown in Table 5 [36,37]. The *i*_corr_ values obtained using Equation (1) were used to determine the corrosion rate (CR) of the steels embedded in the concrete mixtures. The criteria used to analyze the *i*_corr_ results were based on the state of corrosion of carbon steel in Portland cement-based concrete reported in the literature [41], as shown in Table 6.

## 3. Results and Discussion

### 3.1. Corrosion Potential

**Figure 2:**
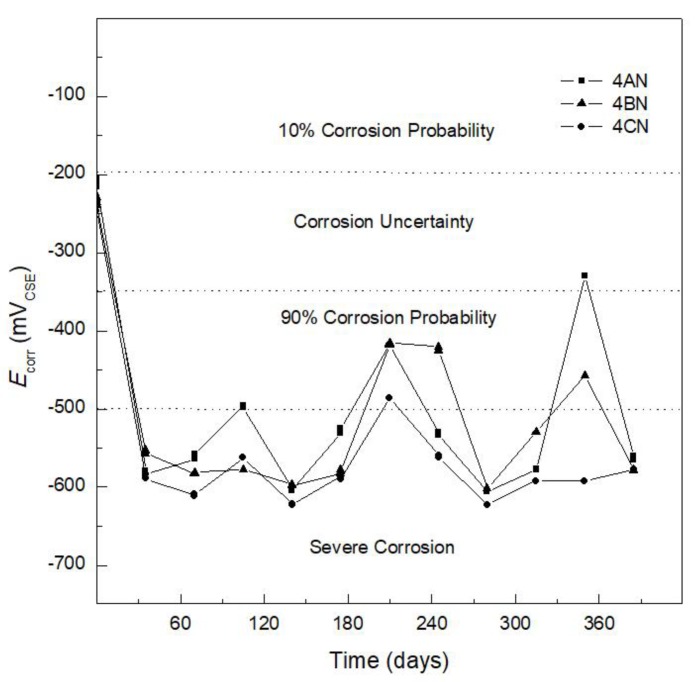
Corrosion potential (*E*_corr_) versus time for AISI 1018 carbon steel embedded in 100% composite Portland cement (CPC) (specimen 4AN), 80% CPC + 20% silica fume (SF) (specimen 4BN), and 80% CPC + 20% fly ash (FA) (specimen 4CN).

Figure 2 shows *E*_corr_ versus exposure time of AISI 1018 CS embedded in concrete for specimen 4AN (100% CPC) (control specimen), specimen 4BN (80%CPC + 20% SF), and specimen 4CN (80% CPC + 20% SF). The evolution of *E_corr_* with time for 4AN (control specimen) was analyzed according to the ASTM C 876-15 standard (see Table 5) [36,37]. Thus, the *E*_corr_ values throughout the exposure time were in the severe corrosion region, with values from −580 mV to −480 mV vs. CSE from day 60 to 180. At day 210, an *E*_corr_ value of −415 mV vs. CSE was reported, and, during the last weeks (365 days), *E*_corr_ tended toward a more active value, −550 mV vs. CSE, in agreement with the literature [42], reporting that reinforced concrete exposed to complete or partial immersion in 3.5% NaCl solution presents *E*_corr_ values between −550 mV and −600 mV vs. CSE from day 50 to 275. It is noted that, in the referenced study, a concrete specimen with 15% FA was evaluated [42], presenting an *E*_corr_ value of −400 mV vs. CSE for 125 days of experimentation. 

It was considered that the corrosion criteria (*E*_corr_) indicated in Table 5 could be used to analyze the different systems in the present study and an AISI 304 SS reinforcement. Figure 2 shows that *E*_corr_ values were in the range of −250 mV to −600 mV vs. CSE from the first week up to 365 days of immersion in 3.5 wt.% NaCl solution. The samples made with AISI 1018 CS and SF (specimen 4BN) or FA (specimen 4CN) showed severe corrosion and similar behavior to those made with 100% CPC (specimen 4AN), with *E*_corr_ values ranging from −600 mV to −450 mV vs. CSE from day 60 to 210. For the final weeks (360 days), *E*_corr_ values remained between −380 mV and −480 mV vs. CSE for specimens 4AN and 4BN (not presenting severe corrosion) and at a value of −600 mV vs. CSE for specimen 4CN (indicating severe corrosion). At the end of the experiment (385 days), the three specimens showed the probability of severe corrosion.

**Figure 3:**
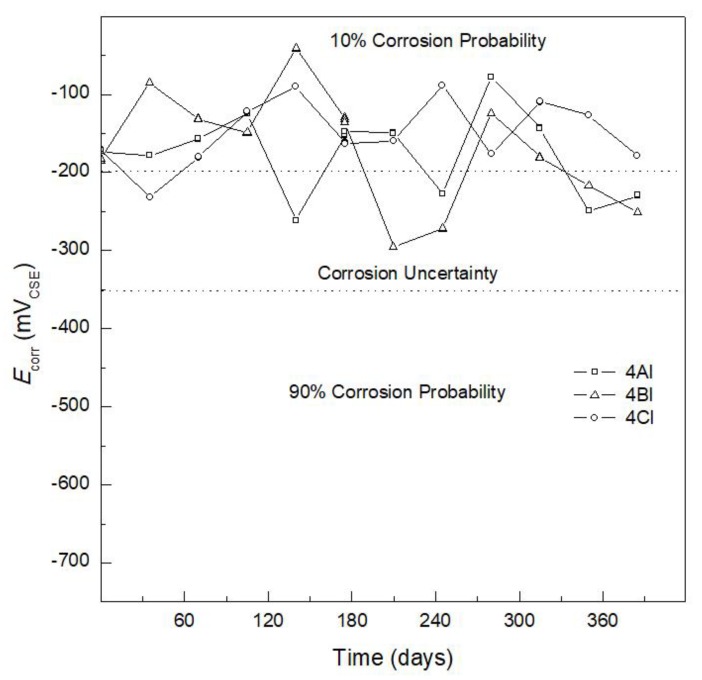
Corrosion potential (*E*_corr_) versus time for AISI 304 stainless steel embedded in 100% CPC (specimen4AI), 80% CPC + 20% SF (specimen 4BI), and 80% CPC + 20% FA (specimen 4CI).

Concrete specimens reinforced with AISI 304 SS (Figure 3) made with 100% CPC (specimen 4AI), 80% CPC + 20% SF (specimen 4BI), and 80% CPC + 20% FA (specimen 4CI) showed that, during the first weeks (curing step) until day 180, the three specimens presented a 10% probability of corrosion, with *E*_corr_ > −200 mV vs. CSE. Specimen 4BI presented an uncertain corrosion probability from day 210 to 245 with *E*_corr_ values between −300 mV and −286 mV vs. CSE, associated with a period of uncertain corrosion probability or small rupture (initiation of pitting corrosion) of the passive layer [43,44,45,46,47]. From day 300 to 360, a passivation process was generated, with *E*_corr_ vales of −200 mV vs. CSE. The *E*_corr_ values for specimen 4CI with 80% CPC + 20% FA presented a more stable *E*_corr_ value than specimens 4AI and 4BI, from day 30 to 365, where their *E*_corr_ values remained in a range from −120 mV to −190 mV vs. CSE, indicating a 10% probability of corrosion.

Crouch et al. [48] stated that one of the most attractive properties of FA is its influence on the improvement of durability, which is the result of the reduction in calcium hydroxide, the most soluble of the hydration products, and changes in the pore solution. In the case of specimen 4BN with 20% SF, the protection provided to the reinforcing steel against corrosion may be attributed to the SF, which reacts with calcium hydroxide released during the hydration of the cement and forms additional hydrated calcium silicate (C–S–H), which improves the durability and mechanical properties of the concrete [49]. Comparison of the *E*_corr_ values in Figure 2 and Figure 3 indicates that a difference in *E*_corr_ behavior can be observed, with the AISI 1018 CS reinforced specimens (4AN, 4BN, and 4CN) (Figure 2) exhibiting non-efficient corrosion resistance regardless of the addition of SF or FA. These specimens had more active potentials than −500 mV vs. CSE, indicating 90% probability of corrosion and severe corrosion. In contrast, the concrete specimens reinforced with AISI 304 SS (4AI, 4BI, and 4CI) (Figure 3) had *E*_corr_ values corresponding to a 10% probability of corrosion and uncertainty of corrosion.

### 3.2. Corrosion Kinetics

**Figure 4:**
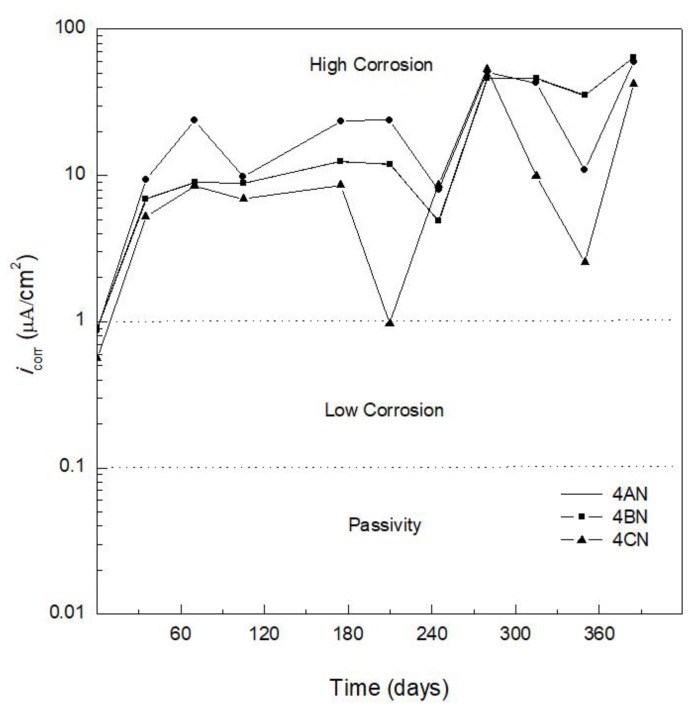
Corrosion current density (*i*_corr_) versus time for AISI 1018 carbon steel embedded in 100% CPC (specimen 4AN), 80% CPC + 20% SF (specimen 4BN), and 80% CPC + 20% FA (specimen 4CN).

**Figure 5:**
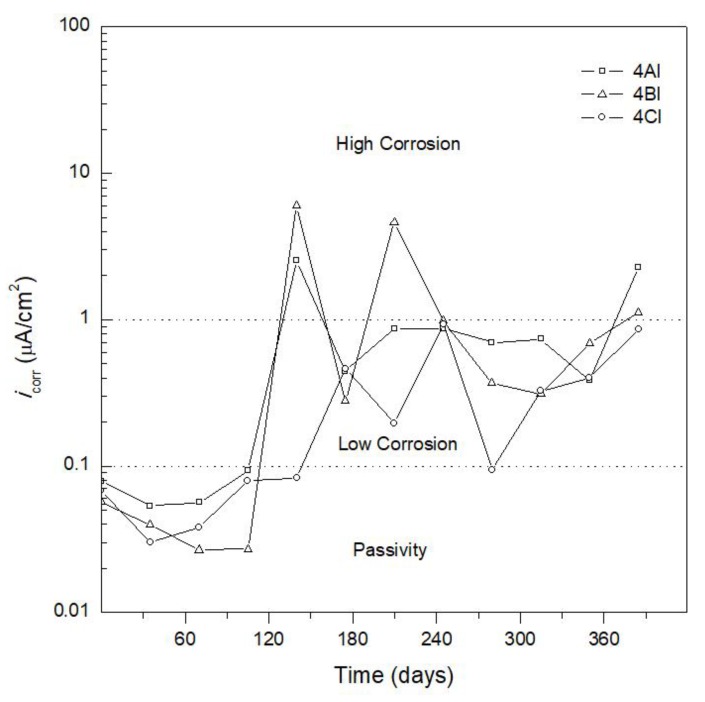
Corrosion current density (*i*_corr_) versus time for AISI 304 stainless steel embedded in 100% CPC (specimen 4AI), 80% CPC + 20% SF (specimen 4BI), and 80% CPC + 20% FA (specimen 4CI).

Figure 4 and Figure 5 show *i*_corr_ versus exposure time. Figure 4 shows AISI 1018 CS embedded in concrete for specimen 4AN (100% CPC) (control specimen), specimen 4BN (80% CPC + 20% SF), and specimen 4CN (80% CPC + 20%FA). The *i*_corr_ values obtained using Equation (1) could be used to obtain the corrosion rate (CR). As indicated above, the criteria used to analyze the *i*_corr_ results for specimen 4AN (control specimen) (Figure 4) were based on the state of corrosion of carbon steel in Portland cement-based concrete reported in Reference [41], as shown in Table 6. It was considered that the corrosion criteria indicated in Table 6 could be used to interpret the *i*_corr_ of the different systems in the present study, for partial replacement of CPC by SF or FA, and for AISI 304 SS reinforcement. 

Figure 4 shows *i*_corr_ results for the three types of specimens reinforced with AISI 1018 CS. During the curing period, *i*_corr_ values from 1 to 3 μA/cm^2^ could be observed. In general, it was seen that the medium was highly aggressive for specimens 4AN and 4BN, with *i*_corr_ values from 3 to 8 μA/cm^2^ up to day 105, decreasing to 0.8 μA/cm^2^ for specimen 4BN at day 210 because of the passivation of the AISI 1018 CS reinforcement. Specimen 4AN presented a decrease of *i*_corr_ from 12 to 3 μA/cm^2^ from day 140 to 245.

The specimen containing 20% FA (specimen 4CN) showed a tendency toward more active *i*_corr_ values than specimens 4AN and 4BN until day 280, where the corrosion behavior was similar to that of specimen 4AN, with values above 10 μA/cm^2^ indicating high corrosion. Concrete specimens reinforced with the AISI 1018 CS presented critical *i*_corr_ values above 10 μA/cm^2^; this corrosive aggressiveness to reinforced concrete was also demonstrated in soils with a concentration of 3 wt.% NaCl, presenting, for the reinforcing steel AISI 1018 CS, values of *i*_corr_ between 3.3 μA/cm^2^ and 3.6 μA/cm^2^ after 260 days of exposure to the soil environment [50].

Figure 5 shows *i*_corr_ results for AISI 304 SS embedded in concrete for specimen 4AI (100% CPC), specimen 4BI (80% CPC + 20% SF), and specimen 4CI (80% CPC + 20% FA). The *i*_corr_ results for AISI 304 SS showed lower values than for the AISI 1018 CS reinforced specimens (see Figure 4). The specimen made with concrete containing 100% CPC (specimen 4AI) had an *i*_corr_ of less than 0.1 μA/cm^2^ until day 105; then, at day 140, it was depassivated with an *i*_corr_ of 2 μA/cm^2^. From day 175 to 350, it was in the passive state, presenting *i*_corr_ values lower than 1 μA/cm^2^, thus indicating a low corrosion level. It was reported that the passive film formed on AISI 304 SS consists of a duplex layer structure, with an inner layer of chromium oxide covered by an iron-oxide outer layer [43,44,47]. In the alkaline environment of the present study, the high Ni content of the AISI 304 SS reinforcement (8.13 wt.%) may have contributed to the corrosion resistance [46]. 

In the last monitoring period (365–385 days), specimen 4AI manufactured with AISI 304 SS presented an *i*_corr_ value of 3 μA/cm^2^ (see Figure 5), showing a benefit in accordance with Bautista et al. [51], indicating that the use of stainless steel in environments with high chloride content is one of the only viable options for achieving more durable reinforced concrete structures. Knudsen et al. [52] also noted that the intelligent use of stainless steel is advisable to repair damaged structures in coastal zones and is a cost-effective option. For a conventional building of 40 flats (~80 m^2^ each) the use of AISI 304 SS instead of the conventional AISI 1018 CS had an additional structure cost of the order of 5–10% [53].

Specimen 4BI with 20% SF showed better corrosion behavior than specimen 4AI manufactured with 100% CPC, with *i*_corr_ values of 0.03 μA/cm^2^ until day 105 and less than 1 μA/cm^2^ from day 280 to 350. Specimen 4CI containing 20% FA exhibited similar corrosion behavior to specimen 4BI, with lower *i*_corr_ values during the monitoring period compared to the specimen made without addition of admixtures (specimen 4AI). A comparison between the specimens reinforced with AISI 1018 CS and AISI 304 SS (see Figure 4 and Figure 5), embedded in concrete without addition and with a mixture of 20% SF or FA, indicates that, after 385 days of immersion in 3.5 wt.% NaCl solution, there was a benefit in terms of corrosion behavior in using the specimens reinforced with AISI 304 SS rather than with AISI 1018 CS. This benefit was further increased by partially replacing CPC with SF or FA, whereby the specimens made with SF showed better corrosion behavior.

## 4. Conclusions

The three specimens made with and without replacement of CPC by SF or FA (SCMs) reinforced with AISI 1018 CS (specimens 4AN, 4BN, and 4CN) showed *E*_corr_ values indicating severe corrosion for 365 days of experimentation. The *i*_corr_ values were higher than 3 μA/cm^2^ for the specimens made with SF and FA (specimens 4BN and 4CN), while the specimen without the addition of mineral additives (specimen 4AN) showed values higher than 10 μA/cm^2^. Given this high *i*_corr_ value, it is considered that SF and FA afford no significant corrosion protection for AISI 1018 CS under the conditions of the present research. The use of supplementary cementitious materials (SCMs) such as silica fume (substitution in 20% of CPC) could contribute to the reduction of the use of Portland cement (PC).

The specimens manufactured with SF and AISI 304 SS reinforcement presented *E*_corr_ values that indicated a 10% probability corrosion, and *i*_corr_ values that indicated passivity of the reinforcement in the first 105 days. Values of *i*_corr_ from 105 to 365 days showed a low corrosion, meaning that the critical chloride threshold value was not reached, where SF and FA have a strong influence due to their capability to decrease the concrete porosity, thus lowering the permeability of chloride ions. The concrete specimens reinforced with AISI 304 SS and immersed in 3.5 wt.% NaCl solution showed better corrosion protection than those with AISI 1018 CS reinforcements. The benefit of using AISI 304 SS was increased by partially replacing CPC with SF or FA, and the specimens made with SF afforded better corrosion behavior. These mixtures can be considered as durable green reinforced concrete due to the corrosion protection they presented in comparison to the specimens reinforced with AISI 1018 CS. In conclusion, the 20% replacement of CPC by SF or FA in concrete exposed to a marine environment and reinforced with AISI 304 SS increased the RCS durability.

## Figures and Tables

**Table 1:** Physical characteristics of the aggregates.

Aggregates	Relative Density (Specific Gravity)	Bulk Density (Unit Weight) (kg/m^3^)	Absorption (%)	Fineness Modulus	Maximum Aggregate Size (mm)
Coarse (gravel)	2.32	1391	5.45	- - -	19
Fine (sand)	2.66	1237	1.97	2.62	- - -

**Table 2:** Proportioning of three concrete mixtures 1 m^3^ (F’c = 35.7 MPa).

Materials	CPC 30R, kg (4AN, 4AI)	Silica Fume (SF), kg (4BN, 4BI)	Fly Ash (FA), kg (4CN, 4CI)
Cement	410	328	328
Partial substitute	0	82	82
Water	205	205	205
Coarse aggregate	890	890	890
Fine aggregate	838	838	838

**Table 3:** Physical and mechanical properties of concrete mixture.

Test	CPC 30R (4AN, 4AI)	Silica Fume (SF) (4BN, 4BI)	Fly Ash (FA) (4CN, 4CI)
Temperature, °C	24.0	21.7	22.4
Slump, cm	4	3	3
Density, kg/m^3^	2150	2188	2173
Compressive strength (F’c), MPa (28 days)	35.9	37.1	36.6

**Table 4:** Chemical composition (wt.%) of the reinforcements tested, AISI 1018 carbon steel, and AISI 304 stainless steel.

Steel	Mass, %									
	C	Si	Mn	P	S	Cr	Ni	Mo	Cu	Fe
AISI 1018	0.20	0.22	0.72	0.02	0.02	0.13	0.06	0.02	0.18	Balance
AISI 304	0.04	0.32	1.75	0.03	0.001	18.20	8.13	0.22	0.21	Balance

**Table 5:** Probability of corrosion according to the measured corrosion potential (*E*_corr_, mV_CSE_) versus a Cu/CuSO_4_ reference electrode (CSE), for reinforced CPC concrete, using an AISI 1018 carbon steel reinforcement [36,37].

Corrosion Potential, *E*_corr_ (mV_CSE_)	
>−200	10% probability of corrosion
−350 < *E*_corr_ < −200	Uncertainty corrosion
−350 < *E*_corr_ < −500	90% probability of corrosion
<−500	Severe corrosion

**Table 6:** Level of corrosion in accordance with the corrosion current density (*i*_corr_) [41].

Corrosion Rate, *i*_corr_ (µA/cm^2^)	Corrosion Level
<0.1	Negligible (passivity)
0.1 < *i*_corr_ < 0.5	Low corrosion
0.5 < *i*_corr_ < 1	Moderate corrosion
>1	High corrosion
